# Morbidity pattern amongst elderly patients presenting at a primary care clinic in Nigeria

**DOI:** 10.4102/phcfm.v3i1.211

**Published:** 2011-04-11

**Authors:** Lawrence A. Adebusoye, Modupe M. Ladipo, Eme T. Owoaje, Adetola M. Ogunbode

**Affiliations:** 1 General Outpatients Department, University College Hospital, Nigeria; 2 Department of Community Medicine, University College Hospital, Nigeria

## Abstract

**Background:**

The elderly comprise the fastest-expanding age group globally, with the greatest increase occurring in developing countries. Disease and deteriorating health are implicitly assumed to be associated with ageing, as chronic medical illnesses mostly present with increasing age.

**Objectives:**

To describe the morbidity pattern of elderly patients presenting at the General Outpatients Clinic of the University College Hospital, Ibadan, Nigeria.

**Method:**

This was a cross-sectional descriptive study of 500 elderly respondents who presented at the clinic between September 2004 and April 2005. They were interviewed according to the format of the electronic, second revision of the International Classification of Primary Care (ICPC-2-E) questionnaire. Main outcome measurements were the prevalence of various morbidities, self-reported health status and socio-demographic characteristics. Body mass index (BMI) was used to assess respondents’ nutritional status.

**Results:**

Respondents were found to under-report their actual health problems. The mean ± s.d of self-reported health problems was 1.7 ± 0.9 (range 1–6), while the mean ± s.d of diagnosed morbidities was 2.7 ± 1.4 (range 1–8). The most prevalent morbidities were hypertension (40.0%), cataracts (39.4%) and osteoarthritis (26.8%). The prevalence of anaemia was 8.0% (females = 11.2%; males = 2.6%), and it was significantly associated with gender (*p* = 0.001). Nutritional status indicated a high prevalence of overweight and obesity (51.8%), which was significantly higher amongst the female respondents than the males (*p* = 0.001).

**Conclusion:**

The prevalence of chronic medical illnesses was high amongst the elderly in this setting. In addition, the elderly under-reported their actual health problems. The high prevalence of overweight and obesity amongst the elderly in this setting calls for public health action that advocates lifestyle changes to manage the health of the elderly.

## Introduction

The elderly comprise the fastest-growing age group globally, with a steep increase seen in developing countries. There is an implicit assumption that disease and deteriorating health are associated with ageing,^1^ although studies on elderly people have debunked this assumption.^2^ Various studies have shown that most elderly people maintain good health until advanced age. Africa has the lowest average life expectancy (51.4 years)^3^ compared to regions like Asia (66.3 years), Latin America (69.2 years), Europe (73.3 years) and North America (76.9 years).^4^ Current estimates indicate that the elderly constitute 6% of Nigeria's population.^5^

Changing demographic transition stages have affected the population of the elderly worldwide. Developed countries tend to be at the fourth stage of demographic transition, where both the birth rate and the death rate are low and population growth slows or even stops, resulting in a large number of elderly people within a dwindling population.^3^ In contrast, the sub-Saharan region, including Nigeria, is generally still at the second stage of demographic transition, where the death rate begins to drop amidst a birth rate that remains high. This leads to a growing population with a longer life expectancy and thus a larger population of elderly people. This demographic transition occurred over a long period in developed countries, but only a short period of time in sub-Saharan Africa. The increasing elderly population in sub-Saharan Africa has led to an increased demand for health and social services.^3^

The elderly usually suffer from multiple pathologies. Clausen et al.^1^ found an average of 5.2 health problems per elderly person living in the Mmankgodi village, Botswana. Other characteristics of the morbidity pattern amongst the elderly are the presence of co-morbidities, non-specific presentation of diseases, impaired drug metabolism and deranged social factors.^1^ Ogunniyi et al.^6^ found that amongst the Idikan community in Ibadan, 59.4% of the elderly population studied had poor or reduced health. The main health problems were hypertension (29.0%), visual impairment (12.1%), cataracts (8.1%), osteoarthritis (6.7%) and neurological problems (5.2%).^6^

With an increasing elderly population in Nigeria, better documentation of their health profiles is needed to inform policy makers of the health problems which they present with. At present geriatrics has not been fully established as a specialty in Nigeria and there is little information about the morbidity pattern of the elderly to form the basis of any meaningful plan of action to improve the quality of life of this section of the population. Furthermore, most studies regarding the morbidity pattern of the elderly in Nigeria have been community based.^6^ Hospital-based data from primary care settings are required for effective planning of health services for the rapidly growing elderly population.

## Ethical consideration

Approval for the study was obtained from the Head of the General Outpatients Department at UCH and the joint University of Ibadan–UCH Ethical Review Board. Each respondent gave informed consent for participation in the study before examination and administration of the questionnaire.

## Methods

### Materials

The study included 500 male and female patients aged 60 years and older who presented at the General Outpatients Clinic at the Univeristy College Hospital (UCH), Ibadan. The age of the respondents was determined by direct recall, association with historical events, the age at marriage and the age at birth of their first child. The respondents were recruited from September to November 2004 (rainy season), and again from February to April 2005 (Harmattan season). This made the seasonal comparison of some morbidities possible. On average, 892 new adult patients were seen at the clinic per month, of whom elderly patients constituted 112 (12.6%).

### Setting

This study was conducted at the General Outpatients Clinic of the UCH, Ibadan, the capital city of Oyo state, Nigeria. Founded in 1957, UCH is a tertirary academic institution with 1000 beds. Patients from across Nigeria and the West-African sub-region are referred to UCH. The General Outpatients Clinic serves as a primary care clinic within a tertiary hospital setting, as most patients seen at UCH are managed at first contact, and very few are subsequently referred to specialty units and paramedical services within UCH. The clinic is run by consultant family physicians and postgraduate resident doctors in Family Medicine.

### Design

This was a cross-sectional descriptive study which assessed the morbidity pattern of elderly people who were recruited using universal sampling. Respondents who met the inclusion criteria were recruited every morning from the triaging (sorting) hall as they presented at the clinic. A technologist from the laboratory department administered the questionnaire to all consenting respondents. A urine and a packed cell volume (PCV) blood sample were collected from each respondent in, respectively, a labelled universal bottle and heparinised capillary tubes immediately afterwards. Before the departure of a respondent, the questionnaire was checked for completeness and the urine and the blood samples were checked for correctness with regard to the respondent's identification number.

### Procedure

The respondents were interviewed using a structured questionnaire based on the electronic, second version of the International Classification of Primary Care (ICPC-2-E) questionnaire as developed by the World Organization of Family Doctors.^7^ The questionnaire has previously been used in an African study.^1^ The ICPC-2-E assesses health problems related to (1) general signs and symptoms, (2) blood, (3) digestive system, (4) eyes, (5) ears, (6) circulatory system, (7) musculoskeletal system, (8) mental illness, (9) neurology, (10) respiratory system, (11) skin, (12) endocrine, metabolic and nutritional functions, (13) urinary system, (14) female genital, and (15) male genital system.

During this study the questionnaire sought information on the respondents’ socio-demographic data as well as other information regarding self-reported health problems (presenting complaints) and doctor-evaluated health problems. Laboratory analysis of PCV and urinalysis were performed for every respondent. Urinalysis is the cheapest and most commonly used method to screen for diabetes mellitus at the primary care level in Nigeria, while PCV analysis was used to detect anaemia.

For anthropometric measurements height was measured to the nearest centimetre using a stadiometer (Seca, Hanover, USA). Weight was measured to the first decimal (kg) using a weighing scale [Hana, Shenzhen]. The zero mark was checked after every reading for accuracy. The body mass index (BMI) was calculated as weight/height^2^. BMI values of lower than 18.4 were defined as underweight, 18.5–24.9 as normal, 25.0–29.9 as overweight and higher than 30.0 as obese.^8^

Blood pressure was measured with an Accoson mercury sphygmomanometer (Accoson, Essex), which had been calibrated and validated before use. The patients were seated comfortably with their left arm exposed and supported at the level of the heart and their feet on the floor. Patients were allowed to relax and measurement started after 5 minutes’ rest. Appropriate cuff sizes were used for each patient, encircling at least 80% of the arm. The appearance of the first Korotkoff sound was taken as the systolic blood pressure and the diastolic blood pressure was recorded at the disappearance of the fifth Korotkoff sound. Two readings, separated by 2 min, were averaged for the reported blood pressure measurement.^9^ Stage 1 hypertension was defined as

systolic blood pressure of 140–159 mmHg and diastolic blood pressure of 90–99 mmHg, while stage 2 was a systolic blood pressure higher than 160 mmHg and diastolic blood pressure higher than 100 mmHg.^10^ PCV below 30% was taken as an indication of anaemia. Some of the respondents were referred to other specialist clinics within the facility for definitive diagnoses.

The British Registrar General's classification was used for classification of social class.^11^

### Statistical analysis

The questionnaires were cross-checked after each interview and coded serially. SPSS (version 15) was used for data entry, cleaning and analysis. Descriptive statistics were used to describe socio-demographic characteristics of the respondents. Appropriate charts were used to illustrate categorical variables. Chi-square statistics were used to assess association between categorical variables. Statistical significance was set at *p ≤* 0.05

## Results

The socio-demographic characteristics of the study sample are shown in table1. Of the 500 elderly respondents, 311 (62.2%) were female and 189 (37.8%) were male. This gave a female: male ratio of 1.7:1. Their mean age was 67.8 years (s.d = 7.1 years, range 60–104 years). The modal age group was 60–69 years. The monthly income of the respondents ranged from 2000 naira to 175 000 naira (equivalent to $13.33–$1166.67), with a median income of 5000 naira, which was much lower than the Nigeria's minimum wage of 11 500 naira. The majority of respondents (*n* = 295, 59.0%) were married, 170 (34.0%) were widowed, 24 (4.8%) were separated from their spouses, 10 (2.0%) were divorced and one respondent was single. More than half the respondents (*n* = 274, 54.8%) were still employed. Almost half of the respondents (*n* = 226, 45.2%) had their children and/or grandchildren living with them, while 13.8% were currently living with their spouses in their own home. The majority of the respondents (52.4%) lived below the World Bank's poverty line of $1 a day.^12^

**TABLE 1:** Socio-demographic characteristics of respondents by gender distribution.

Characteristic	Male		Female		Total	
		
	*n*	%	*n*	%	*N*	%
**Age (years)**						
60–64	57	30.2	119	38.3	176	35.2
65–69	44	23.3	82	26.3	126	25.2
70–74	51	27.0	62	20.0	113	22.6
75–79	20	10.6	20	6.4	40	8.0
80–84	15	7.9	14	4.5	29	5.8
≥ 85	2	1.0	14	4.5	16	3.2
**Marital status**						
Married	153	81.0	142	45.7	295	59.0
Single	1	0.5	0	0.0	1	0.2
Divorced	4	2.1	6	1.9	10	2.0
Widowed	20	10.6	150	48.2	170	34.0
Separated	11	5.8	13	4.2	24	4.8
**Social class**						
I	5	2.7	0	0.0	5	1.0
II	31	16.4	21	-6.8	52	10.4
III	32	16.9	2	0.6	34	6.8
IV	49	25.9	31	10.0	80	16.0
V	72	38.1	259	82.6	329	65.8
**Income**						
Below poverty line (< $1/day)	78	41.3	184	59.2	262	52.4
Above poverty line (> $1/day)	111	58.7	127	40.8	238	47.6
**Number of children alive**						
1–2	9	4.8	33	10.6	42	8.4
3–4	30	15.9	110	35.4	140	28.0
≥ 5	150	79.3	168	54.0	318	63.6
**Living arrangement**						
Alone	10	5.3	44	14.1	54	10.8
With spouse in own home	24	12.7	45	14.5	69	13.8
With children or grandchildren in own home	140	74.1	86	27.7	226	45.2
With children or grandchildren in their home	8	4.2	110	35.4	118	23.6
With other relatives	7	3.7	26	8.3	33	6.6
**Family support**						
Self	80	42.3	45	14.5	125	25.0
Spouse	2	1.1	9	2.9	11	2.2
Children or grandchildren	100	52.9	248	79.7	348	69.6
Other relatives	4	2.1	8	2.6	12	2.4
Friends	3	1.6	1	0.3	4	0.8

*Source*: World Bank 2004.^12^

*n*, number.

Based on the ICPC 871 health problems were self-reported, with an average of 1.7 health problems per respondent (range 1–6). The majority of problems (*n* = 206, 41.2%) related to general body symptoms like fever, body pains and malaise. Almost an equal number of neurological and musculoskeletal problems were reported (*n* = 132, 26.4% and *n* = 130, 26.0% respectively). In addition, 99 respondents(19.8%) reported eye problems such as pain, redness and poor vision (Table 2).

**TABLE 2:** Self-reported health problems of respondents using ICPC classification.

ICPC classification	Number of presenting complaints	

	*n*	%
General body symptoms	206	41.2
Blood	3	0.6
Digestive	78	15.6
Eye	99	19.8
Ear	7	1.4
Cardiovascular	30	6.0
Musculoskeletal	130	26.0
Neurology	132	26.4
Mental illness	53	10.6
Respiratory	51	10.2
Skin	24	4.8
Endocrine, metabolic, nutrition	26	5.2
Urinary	30	6.0
Genital	2	0.4

*n*, number.

**FIGURE 1:**
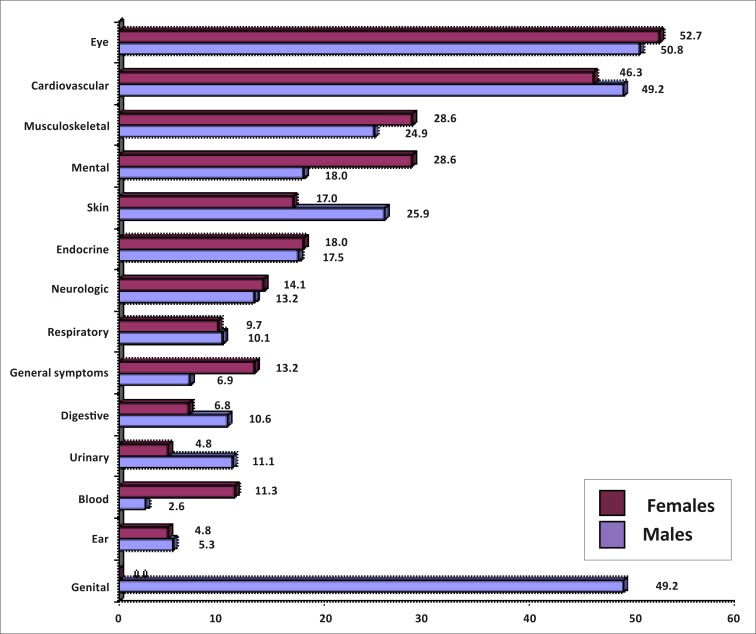
Morbidities by gender distribution using ICPC diagnostic classification.

A total of 1349 morbidities were diagnosed amongst the group, with an average of 2.7 per respondent (range 1–8). Figure 1 illustrates the morbidities according to the ICPC, organised according to gender. The most prevalent morbidities were found in the eyes (males = 50.8%; females = 52.7%), and cardiovascular (males = 49.2%; females = 46.3%) and musculoskeletal systems (males = 24.9%; females = 28.6%). Erectile dysfunction was the only cause of genital morbidity in the male respondents. Distribution across specific morbidities is shown in Table 3. Hypertension (40.0%) was the most commonly observed morbidity, followed by cataracts (39.4%) and osteoarthritis (26.8%).

**TABLE 3:** Morbidity pattern among respondents.

Morbidity	*N* = 500	

	*n*	%
Hypertension	200	40.0
Cataracts	197	39.4
Osteoarthritis	134	26.8
Skin ulcers (wounds)	75	15.0
Diabetes	66	13.2
Psychosomatic disorders	65	13.0
Depression	51	10.2
Malaria	41	8.2
Anaemia	40	8.0
Heart failure	37	7.4
Vertigo	33	6.6
Pterygium	28	5.6
Upper respiratory tract Infection	26	5.2
Hearing impairment	25	5.0
Goitre	23	4.6
Seizure disorders	19	3.8
BPH	16	3.2
Glaucoma	15	3.0
Peptic ulcer disease	15	3.0
Lipoma	13	2.6
Malignancies	11	2.2
Eczema	11	2.2
Cerebrovascular disease	11	2.2
Corneal opacity	11	2.2
Tuberculosis	9	1.8
Chronic obstructive pulmonary disease	9	1.8
Chronic renal insufficiency	8	1.6
Chronic liver disease	8	1.6
Psychosis	7	1.4
Gastroenteritis	6	1.2
Urinary tract infection	6	1.2
Asthma	5	1.0
Allergic conjunctivitis	5	1.0
Viral conjunctivitis	4	0.8
Fissure in ano	3	0.6
Haemorrhoids	3	0.6
Cellulitis	3	0.6
Hernia	3	0.6
Head injury	3	0.6
Hepatitis	2	0.4
Bell's palsy	2	0.4
HIV/AIDS	2	0.4
Erectile dysfunction†	93	49.2
Others	5	1.0

Some respondents presented with multiple morbidities.

*n*, number; BPH, Benign prostatic hyperplasia.

† Erectile dysfunction only occurs in men.

Anthropometric measurement of the respondents showed their mean height ± s.d to be 1.6 ± 0.1 m (range = 1.2–1.8 m) and mean weight ± s.d to be 63.5 ± 13.5 kg (range = 30.0– 109.0 kg). At a mean height of 1.62 ± 0.07 m the men were significantly taller than the women, whose mean height was 1.53 ± 0.06 m (*p* = 0.001; *t* = 15.543). However, mean weight of men and women (63.47 ± 12.12 kg vs 63.47 ± 14.32 kg) was not significantly different (*p* = 0.998; *t* = –0.002). The mean BMI of the respondents was 25.9 ± 5.5 (range = 13.3–47.2), which was significantly higher amongst women (27.1 ± 5.7) than men (24.1 ± 4.6) (*p* = 0.001; *t* = 5.876). According to BMI cut-off values, the majority (*n* = 205, 41.0%) were described to have a normal BMI, 160 (32.0%) were overweight, 106 (21.2%) were obese and 29 (5.8%) were underweight. More men than women were underweight (63.3% vs 36.7%). Conversely, more women than men were obese (82.7% vs 17.3%). There was a significant association between BMI and gender (*χ*^2^ = 31.003, *p* = 0.001).

Laboratory investigation showed the overall prevalence of anaemia to be 8.0%, which was significantly higher among women (11.2%) than men (2.6%) (χ^2^ = 13.011, *p* = 0.001). The mean PCV was 35.6 ± 4.6% (range = 10.0–50.0%); 37.8 ± 4.3% amongst men and 34.2 ± 4.3% amongst women. The urinalysis showed that 8.8% of respondents had glycosuria and 17.8% had proteinuria.

## Discussion

### Outline of results

This study highlighted the morbidity pattern of the elderly patients presenting at a general outpatient clinic in Nigeria.

There was a predominance of female respondents who outnumbered their male counterparts by 1.7 to 1. This may be attributed to life expectancy, which is higher for women than men. At the time of the study, the life expectancy of Nigerian women was 47 years compared to 46 years for men.^13^ Also, women visit clinics for more frequently than men.

The average number of self-reported health problems (presenting complaints) was less than the average number of morbidities (diagnoses) found amongst the respondents. This shows that the elderly often under-report their health problems and may attribute certain health problems to ageing, thus finding it unnecessary to complain about them to a physician. Finding multiple morbidities per respondent was similar to findings of earlier studies, although fewer were found than for studies in Botswana and India.^1, 14^ The average number of morbidities (diagnoses) found amongst the respondents (2.7 morbidities) was less than those reported for Botswana (5.2 morbidities) and India (6.0 morbidities).^1, 14^ These disparities could be related to cultural perception of illnesses and global differences in the prevalence of diseases.^1, 6^ Thus, the importance of a detailed history, comprehensive examination and necessary investigations cannot be emphasised enough in the management of elderly patients.

Eye problems were the most commonly diagnosed morbidity amongst the respondents; cataracts accounted for more than three-quarters of the diagnoses. The high prevalence of cataracts (39.4%) amongst the respondents may be attributed to cultural fear of surgery, the cost of surgery and the belief that the diminution of vision is the consequence of ageing. In addition, elderly people seldom complain of health problems that do not inflict pain^6^ and they may not readily agree to have eye surgery.

Hypertension was registered in two-fifths of the respondents. Globally, studies have shown that the prevalence of hypertension is increasing and may become a major primary health care problem with an increasing elderly population because blood pressure rises with age in nearly all populations.^1, 6, 10, 15, 16^

Musculoskeletal problems were the third most common morbidities found amongst the respondents, with osteoarthritis found amongst 26.8% of the respondents. In previous studies amongst elderly African communities, osteoarthritis was one of the commonly observed problems.^1, 6, 17^ Osteoarthritis compromises mobility and consequently tends to impair social and occupational functioning.^6^ It leads to dependency on others, especially family members.

Problems related to mental health were observed more often amongst the female respondents (28.6%) than the males (18.0%). The mental problems diagnosed included psychosomatic disorders, depression and psychosis. Uwakwe^18^ reported mental illness to occur at a prevalence of 23.1% among elderly Nigerians.

At 13.2% diabetes mellitus was the most common endocrine problem found in this study. This prevalence was higher than what has been reported for the general population in a previous study,^19^ probably because the present study was hospital based and also owing to a higher prevalence of diabetes with advancing age. Thus, the higher prevalence of diabetes in this study might not be surprising.

Overweight and obesity were found in more than half of the respondents. Obesity was significantly more prevalent amongst women than men (*p* = 0.001). Bakare^5^ reported similar findings amongst elderly people in south-western Nigeria. The dietary habits of elderly Nigerians tend towards consumption of high-energy foods like carbohydrates and animal fats. This nutritional habit and minimal physical activity have been implicated as the main reasons for the development of obesity, a situation that is contrary to the recipe for a healthy weight and healthy life.^5^ Anaemia was found in 8.0% of the respondents and was significantly more prevalent among women than men (*p* = 0.001). Nutritional anaemia is common amongst the elderly due to intrinsic physiological decrease in food intake, taste, smell and gastric emptying, and dysregulation of satiation called ‘physiological anorexia of ageing’.^20^ Another common cause of anaemia in the elderly is malignancy, which becomes more prevalent with advancing age.^20^

### Practical implication

The physician's goals in the management of the elderly should include health promotion, early disease detection and prevention of frailty when possible. The traditional screening tests used during evaluation of younger patients should not be withheld from the elderly. The elderly should have routine urinalysis and BMI, PCV and blood pressure measurement.

### Limitation

This was a hospital-based study of which the results may not be applicable to the general population.

### Recommendation

The elderly should be encouraged to undergo periodic medical checks at a clinic for routine appraisal of their health status, so as to allow early detection and treatment of their morbidities. More studies on the morbidities of elderly patients presenting at hospitals in developing countries are needed to formulate a longitudinal frontline health care plan for the elderly.

## Conclusion

This study has demonstrated that the elderly present with multiple morbidities and under-report their health problems, which they often attribute to ageing. The most prevalent health problems of the elderly were chronic medical illnesses like hypertension, cataracts, osteoarthritis and psychosomatic disorders – all conditions that are treatable. The high prevalence of overweight and obesity (found in more than half of the respondents) is worrisome when its public health impact is considered. Thus, physicians should include advice on lifestyle modification in their management of elderly patients.
